# Towards the Holistic Assessment of Scar Management Interventions

**DOI:** 10.3390/ebj3010018

**Published:** 2022-03-08

**Authors:** Jonathan Mathers

**Affiliations:** Institute of Applied Health Research, University of Birmingham, Birmingham B15 2TT, UK; j.m.mathers@bham.ac.uk

**Keywords:** scar management, scar assessment, patient centred, scar treatment experience, psychosocial adaptation

## Abstract

Presently, research assessments of burn scar management interventions focus on measures of scarring and scar features. However, qualitative research demonstrates that patients experience scarring and scar management therapies holistically. Patient-centred assessment should reflect this. An agreement is required regarding what to assess, which tools and measures to use and at what time points. Key issues include (1) whether and how burn- or scar-related quality-of-life measures could be included in the assessment of scar management interventions and how these are weighed against scar measures; (2) routine inclusion of the assessment of treatment burden (or treatment experience) in comparative research and (3) generating further understanding of the relationship between scar management and psychosocial adaptation, along with an assessment of this. A debate concerning a holistic and standardized evaluation of scar management interventions is needed to ensure that future evidence-based decisions are made in a patient-centred manner.

## 1. The Issue

There is a need for better evidence to underpin patients’, parents’ and clinicians’ choices regarding scar therapies in burns aftercare [1]. A consensus is needed regarding what to assess in research studies, which tools and measures to use and at what time points. Presently, clinical research assessments of scar management interventions focus more predominantly on measures of scarring and individual scar features [2,3,4]. Systematic reviews demonstrate that judgements of the efficacy of pressure garment therapy (PGT), one mainstay of scar management, have largely been made according to scar outcomes including the use of aggregate scar scales and individual measures, such as scar thickness, colour and pliability [2,3]. However, patients experience scarring and scar management therapies much more broadly, and a patient-centred evaluation of scar management interventions should reflect this.

Scar management impacts not only on physical scar processes and outcomes, but also psychological and psychosocial processes. Qualitative research with patients and parents of paediatric patients shows that they view scar management and related outcomes holistically [5]. They value a complex set of factors beyond the clinical assessment of scarring, including function, psychological adaptation, body image, activities, relationships and treatment burden. Martin’s phenomenological study of the experience of PGT also shows how scar management impacts people’s self-image and identity [6]. Whilst patients’ accounts of scar management and related outcomes do include descriptions of scarring and features of scars, these are likely influenced by expectations for scar therapies set via interactions with clinical staff. In a UK interview study, patients often used terms that were also used by therapists to describe the clinical intention of PGT; whilst in unprompted discussions around scarring, they tended to make overarching personal and subjective judgements regarding their appearance [5]. Thus, demonstrable effects in measurable scar features, clinically and statistically, may not necessarily result in equivalent effects on patients’ own judgements of scarring and appearance.

A systematic review and synthesis of qualitative evidence in adult burns to define a patient-centred set of outcome domains showed that psychosocial adaptation was a core overriding concern for patients [7]. As there is not always a clear, consistent and strong association between injury or scar severity and psychosocial outcomes [8,9], we should not assume that incremental benefits to scar outcomes translates to longer term psychosocial outcomes that are appreciable to patients.

Qualitative research studies also demonstrate that scar therapies and other treatments are associated with a considerable burden, which in turn not only impact their adherence to therapy [10,11], but also potentially broader psychological and psychosocial outcomes [6]. A detailed understanding of this burden should be a core component of any holistic assessment and comparison of scar interventions.

Taken together, these observations challenge a predominant reliance on narrowly defined assessments of scarring if the evaluation of scar interventions is to be patient-centred.

## 2. What Is Required

A further discussion and debate to understand how to assess the effect of scar management interventions holistically is now needed. In 2020, Edgar and colleagues called for a discussion regarding the best practice in quality-of-life assessment in burns, suggesting that a refocusing on the impact of ‘scarring of the mind’ on recovery of quality of life was needed, as opposed to a sole reliance on scar outcomes [12]. The case presented here is analogous, though with a challenge to the distinction between scar and quality-of-life outcomes. Issues to resolve include:

Firstly, a discussion is needed on whether a health- or scar-related quality-of-life assessment should routinely be included in the evaluation of scar management interventions, and if so, how this is weighed against scar measures and outcomes. In a UK National Institute for Health Research feasibility study for a trial of PGT, one key recommendation was that a measure of burn- or scar-related quality of life be considered as a primary or co-primary endpoint in any subsequent trial [13]. Implementation of this requires an agreement on which tools to use to ensure the standardisation of clinical research assessment, comparability of outcomes across studies and to facilitate a more effective synthesis of evidence of effectiveness. Currently, there are a number of tools available; the BSHS-B is the most commonly used and recommended by Edgar and colleagues [12]; there is also the scar-specific quality-of-life tool, the BBSIP [14], and more recently, the development of the CARe burn scales in the UK [15]. An agreement on which scales to routinely use in the evaluation of scar management interventions is needed, for example, according to conceptual content [5,7], validation evidence [16], availability for adults and children, and also to assess the impact on parents. For young children, the availability and validity of parent proxy measures is also a key issue concerning assessment in paediatric burns. Consideration of cross-cultural issues, including an equivalence of concepts, and availability and validation of tools in relevant languages is needed.

Secondly, treatment burden should be a core component of assessment. At present, whilst burden is assessed to a limited extent as components in some burn-related quality-of-life tools, there is not a comprehensive conceptualisation of treatment burden in burns to inform a detailed assessment of this. The assessment of treatment burden across chronic diseases is still an emerging research field. Attempts have been made to conceptualise treatment burden across conditions, with ongoing efforts to operationalise these as part of clinical assessment [17,18,19]. Whilst the role treatment plays in patients’ self-management strategies in burns have been acknowledged [20], a detailed definition and conceptualisation of treatment burden to underpin assessment is required. Perhaps this could be alternatively conceptualised as ‘treatment experience’ as the label burden has largely negative connotations, whilst we know that some patients experience scar management as a positive and enabling treatment process [5,6].

Thirdly, the relationship between scar management and psychosocial adaptation to burn injury, and the assessment of this, should also be the focus of further research. A recent systematic review and synthesis of qualitative research studies undertaken with adult burns patients showed that adaptation is the most commonly occurring theme in the existing qualitative burns literature, regardless of national and cultural setting [7]. Sense of self was suggested as a core overarching concept that should be considered as part of outcome assessment related to adaptation. Psychosocial adaptation, a term used across disciplines, is often a slippery and ill-defined concept that has been operationalised in a myriad of different ways, for example, with varied terminology, conceptualization and assessment [21]. Adaptation to chronic disease has been studied and theorized in both sociology and psychology; work from medical sociology focused on the threat to self that injury and chronic disease comprise [22,23], and via psychological theories of adaptation, including theories of stress and coping [24,25]. Whilst psychosocial adaptation is complex conceptually, and requires consensus regarding assessment, it is paramount in patients’ narratives of recovery from burn injury. In health research, it is often conflated with health-related quality of life. Presently, some burn-specific quality-of-life measures include content that is arguably reflective of psychosocial adaptation components, but these are not expressly labelled or conceptualised as such. Recently, there have been calls to include psychosocial adaptation in the International Classification of Functioning, Disability and Health [26].

Finally, there should be further reflection on the patient-centred use of scar assessment tools in routine clinical practice. Patient-centred scar assessment has been explored via qualitative research in the UK [27]. Whilst this work may not be generalisable to all settings, it provides a framework to understand patient-centred scar assessment during routine follow-up and some simple recommendations for patient-centred practice, such as a clarity of purpose for scar assessment and clear feedback loops to patients. Participants in this research also noted that they valued the ability to focus on psychological impact and rehabilitation via the use of tools such as the BBSIP. This finding is echoed in plans for innovative work to establish how graphical feedback from e-PROMs might be therapeutic for children with life-altering skin conditions [28].

In conclusion, similar to previous calls to assess quality of life routinely in patient follow-up, a further debate concerning the holistic evaluation of scar management interventions is needed to ensure that future evidence-based decisions are made in a patient-centred manner.

## References

[1] Finnerty C.C., Jeschke M.G., Branski L.K., Barret J.P., Dziewulski P., Herndon D.N. (2016). Hypertrophic scarring: The greatest unmet challenge after burn injury. Lancet.

[2] Anzarut A., Olson J., Singh P., Rowe B.H., Tredget E.E. (2009). The effectiveness of pressure garment therapy for the prevention of abnormal scarring after burn injury: A meta-analysis. J. Plast. Reconstr. Aesthet. Surg..

[3] Ai J.W., Liu J.T., Pei S.D., Liu Y., Li D.S., Lin H.M., Pei B. (2017). The effectiveness of pressure therapy (15–25 mmHg) for hypertrophic burn scars: A systematic review and meta-analysis. Sci. Rep..

[4] Young A.E., Davies A., Bland S., Brookes S., Blazeby J.M. (2019). Systematic review of clinical outcome reporting in randomised controlled trials of burn care. BMJ Open.

[5] Jones L.L., Calvert M., Moiemen N., Deeks J.J., Bishop J., Kinghorn P., Mathers J. (2017). Outcomes important to burns patients during scar management and how they compare to the concepts captured in burn-specific patient reported outcome measures. Burns.

[6] Martin C., Bonas S., Shepherd L., Hedges E. (2016). The experience of scar management for adults with burns: An interpretative phenomenological analysis. Burns.

[7] Mathers J., Moiemen N., Bamford A., Gardiner F., Tarver J. (2020). Ensuring that the outcome domains proposed for use in burns research are relevant to adult burn patients: A systematic review of qualitative research evidence. Burns Trauma.

[8] Lawrence J.W., Mason S.T., Schomer K., Klein M.B. (2012). Epidemiology and impact of scarring after burn injury: A systematic review of the literature. J. Burn Care Res..

[9] Cleary M., Kornhaber R., Thapa D.K., West S., Visentin D. (2020). A quantitative systematic review assessing the impact of burn injuries on body image. Body Image.

[10] Andrews N., Jones L.L., Moiemen N., Calvert M., Kinghorn P., Litchfield I., Bishop J., Deeks J., Mathers J. (2018). Below the surface: Parents’ views on the factors that influence treatment adherence in paediatric burn scar management—A qualitative study. Burns.

[11] Crofton E., Meredith P., Gray P., O’Reilly S., Strong J. (2020). Non-adherence with compression garment wear in adult burns patients: A systematic review and meta-ethnography. Burns.

[12] Edgar D.W., Van Daele U., Spronk I., van Baar M., van Loey N., Wood F.M., Kazis L., Meirte J. (2020). Seeding the value based health care and standardised measurement of quality of life after burn debate. Burns.

[13] Moiemen N., Mathers J., Jones L., Bishop J., Kinghorn P., Monahan M., Calvert M., Slinn G., Gardiner F., Bamford A. (2018). Pressure garment to prevent abnormal scarring after burn injury in adults and children: The PEGASUS feasibility RCT and mixed-methods study. Health Technol. Assess..

[14] Tyack Z., Ziviani J., Kimble R., Plaza A., Jones A., Cuttle L., Simons M. (2015). Measuring the impact of burn scarring on health-related quality of life: Development and preliminary content validation of the Brisbane Burn Scar Impact Profile (BBSIP) for children and adults. Burns.

[15] Griffiths C., Guest E., Pickles T., Hollen L., Grzeda M., White P., Tollow P., Harcourt D. (2019). The Development and Validation of the CARe Burn Scale-Adult Form: A Patient-Reported Outcome Measure (PROM) to Assess Quality of Life for Adults Living with a Burn Injury. J. Burn Care Res..

[16] Griffiths C., Guest E., White P., Gaskin E., Rumsey N., Pleat J., Harcourt D. (2017). A Systematic Review of Patient-Reported Outcome Measures Used in Adult Burn Research. J. Burn Care Res..

[17] Sav A., Kendall E., McMillan S.S., Kelly F., Whitty J.A., King M.A., Wheeler A.J. (2013). ‘You say treatment, I say hard work’: Treatment burden among people with chronic illness and their carers in Australia. Health Soc. Care Community.

[18] Sav A., King M.A., Whitty J.A., Kendall E., McMillan S.S., Kelly F., Hunter B., Wheeler A. (2015). Burden of treatment for chronic illness: A concept analysis and review of the literature. Health Expect..

[19] Sav A., Salehi A., Mair F.S., McMillan S.S. (2017). Measuring the burden of treatment for chronic disease: Implications of a scoping review of the literature. BMC Med. Res. Methodol..

[20] Litchfield I., Jones L.L., Moiemen N., Andrews N., Greenfield S., Mathers J. (2019). The role of self-management in burns aftercare: A qualitative research study. Burns.

[21] Londono Y., McMillan D.E. (2015). Psychosocial adaptation: An evolutionary concept analysis exploring a common multidisciplinary language. J. Adv. Nurs..

[22] Charmaz K. (1983). Loss of self: A fundamental form of suffering in the chronically ill. Sociol. Health Illn..

[23] Morse J.M., O’Brien B. (1995). Preserving self: From victim, to patient, to disabled person. J. Adv. Nurs..

[24] Stanton A.L., Revenson T.A., Tennen H. (2007). Health psychology: Psychological adjustment to chronic disease. Annu. Rev. Psychol..

[25] Helgeson V.S., Zajdel M. (2017). Adjusting to Chronic Health Conditions. Annu. Rev. Psychol..

[26] Dekker J., de Groot V. (2018). Psychological adjustment to chronic disease and rehabilitation—An exploration. Disabil. Rehabil..

[27] Price K., Moiemen N., Nice L., Mathers J. (2021). Patient experience of scar assessment and the use of scar assessment tools during burns rehabilitation: A qualitative study. Burns Trauma.

[28] Tyack Z., Simons M., McPhail S.M., Harvey G., Zappala T., Ware R.S., Kimble R.M. (2021). Improving the patient-centred care of children with life-altering skin conditions using feedback from electronic patient-reported outcome measures: Protocol for a hybrid effectiveness-implementation study (PEDS-ePROM). BMJ Open.

